# The Impact of Prehabilitation on Clinical Outcomes in Liver Surgery: A Systematic Review and Meta‐Analysis

**DOI:** 10.1002/wjs.70501

**Published:** 2026-07-20

**Authors:** Lyrics M. Noba, Robyn J. Stiger, Declan F. J. Dunne, Lawrence D. Hayes, Christopher J. Gaffney, Ramani S. Moonesinghe, Ewen M. Harrison

**Affiliations:** ^1^ School of Healthcare Faculty of Medicine and Health University of Leeds Leeds UK; ^2^ Oxford Institute of Applied Health Research Oxford Brookes University Oxford UK; ^3^ Liverpool University Hospitals NHS Foundation Trust Liverpool UK; ^4^ Faculty of Health and Medicine Lancaster University Lancaster UK; ^5^ Centre for Perioperative Medicine Research Department for Targeted Intervention University College London London UK; ^6^ Usher Institute University of Edinburgh Edinburgh UK

**Keywords:** hepatectomy, length of stay, liver resection, meta‐analysis, postoperative complications, prehabilitation, systematic review

## Abstract

**Background:**

Major liver surgery is associated with significant physiological stress and a high rate of postoperative complications. Prehabilitation aims to enhance physiological reserve before surgery. Despite the growing volume of liver resections worldwide, the impact of prehabilitation on clinical and economic outcomes in patients undergoing elective liver resection is poorly understood.

**Methods:**

A systematic review and meta‐analysis was reported in accordance with PRISMA guidelines. MEDLINE, Embase, Web of Science and the Cochrane Library were searched in November 2025. Randomized controlled trials and comparative observational studies evaluating preoperative programs with a structured exercise component, with or without additional nutritional and psychological support in adult patients undergoing major elective liver surgery were included.

**Results:**

Six studies (four RCTs and two comparative cohort studies) comprising 557 patients were included. Prehabilitation resulted in significantly fewer overall postoperative complications (OR 0.55; 95% CI, 0.37 to 0.84; *p* = 0.005). No significant differences were observed for length of stay (MD −0.38 days; 95% CI, −1.08 to 0.32; *p* = 0.29), major complications (OR 0.79; 95% CI, 0.50 to 1.27; *p* = 0.33), mortality (OR 0.40; 95% CI, 0.05 to 3.24; *p* = 0.39), readmission (OR 0.85; 95% CI, 0.47 to 1.55; *p* = 0.60) and hospitalization costs (MD = −137.13; 95% CI, −642.19, 367.93; *p* = 0.59).

**Conclusion:**

Prehabilitation significantly reduces overall postoperative complications following liver resection. The absence of standardized, liver‐specific interventions limits the determination of effective program design. Future research should prioritize standardised protocols and evaluate post‐hepatectomy functional recovery, patient‐reported outcomes and cost‐effectiveness.

## Graphical abstract

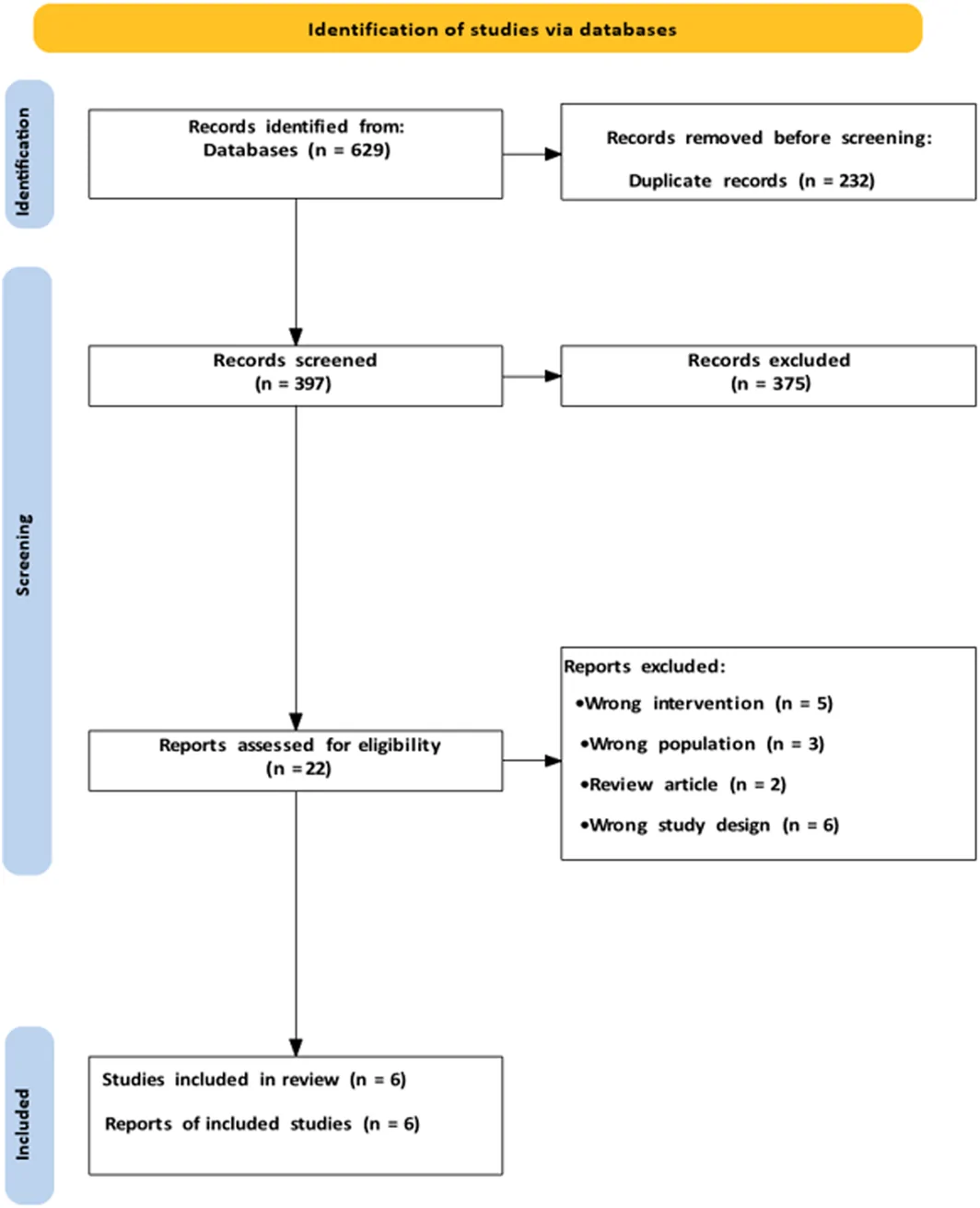

## 1 Introduction

Prehabilitation aims to enhance physiological reserve and functional capacity before surgery, thereby attenuating the surgical stress response and improving surgical outcomes [1, 2]. Evidence supporting prehabilitation in upper gastrointestinal surgery has evolved from predominantly physical activity interventions to more inclusive, multimodal and patient‐centered approaches that combine structured exercise, nutritional optimization and psychological support to enhance patients' functional status before and after surgery [3, 4].

Traditionally, major liver surgery is associated with considerable physiological stress and significant postoperative complication rates [5]. Despite marked improvements in surgical techniques and perioperative care management over recent years, patient frailty and poor baseline fitness remain key factors negatively affecting recovery trajectories and outcomes [6]. The successful implementation of Enhanced Recovery After Surgery (ERAS) in liver surgery has demonstrated clear benefits [7, 8, 9]; however, the role of prehabilitation within this integrated pathway remains poorly defined and varies considerably [8, 10, 11].

The implementation of prehabilitation in liver surgery, specifically, has been hindered by concerns regarding clinical feasibility, safety, patient compliance and the often‐urgent timing of oncological resections [12, 13]. Due to these challenges, the first dedicated studies of structured prehabilitation programs in liver surgery only began to appear in the scientific literature in the mid‐2010s [14, 15].

While several systematic reviews and meta‐analyses have evaluated prehabilitation in hepato‐pancreato‐biliary (HPB) surgery, they often aggregate data from liver and pancreatic procedures despite their distinct physiological insults and recovery pathways [16, 17, 18, 19]. This heterogeneity, combined with the absence of a quantitative synthesis of cost data, limits understanding of the specific impact of prehabilitation in liver resection [20]. To address these gaps, we conducted a systematic review and meta‐analysis evaluating prehabilitation in patients undergoing elective liver resection. Our primary aim was to determine its impact on postoperative complications and length of stay (LOS). Additionally, we performed a quantitative synthesis of available hospitalization cost data to provide preliminary insights into the economic benefit of prehabilitation in liver surgery.

## 2 Methods

This systematic review and meta‐analysis is reported in accordance with the Preferred Reporting Items for Systematic Reviews and Meta‐Analyses (PRISMA) guidelines for meta‐analyses [21]. The protocol was registered on the International Prospective Register of Systematic Reviews (PROSPERO) on the 25th of November 2025 (CRD420251239404).

### 2.1 Search Strategy

A literature search was performed on 27th November 2025 to identify relevant studies. The bibliographic databases searched included MEDLINE, Embase, Web of Science and the Cochrane Library. The full search strategy for each database is provided in Supporting Information S1. The search strategy developed for PubMed included: (“Hepatectomy” OR “iver Resection” OR “Hepatic Surgery” OR “Colorectal Liver Metastases” OR “Hepatocellular Carcinoma”) AND (“Prehabilitation” OR “Preoperative Conditioning” OR “Prehab” OR “Preoperative Exercise”) AND (Standard Care OR Usual Care OR Control Group) AND (Complications OR Morbidity OR Mortality OR “Length of Stay” OR “Hospital Readmission” OR Survival). We also hand‐searched reference lists of included articles and key journals. Conference proceedings were examined, and authors and experts were contacted to identify unpublished studies and to ensure that no relevant studies that met the inclusion criteria were missed.

### 2.2 Eligibility Criteria

Studies were eligible for inclusion if they met the following criteria defined a priori. The population of interest was adult patients (≥ 18 years) undergoing major elective liver surgery. The intervention considered was any preoperative program that included a structured exercise component, with or without additional nutritional and psychological support (e.g., unimodal, bimodal or multimodal prehabilitation). The comparator was standard preoperative or usual care. The primary outcomes were hospital length of stay and postoperative complications. Major complications were defined as Clavien‐Dindo grade III or higher. Secondary outcomes included mortality, readmission rates and hospital costs. We included randomized controlled trials (RCTs) and comparative observational studies. No restrictions were placed on language or publication date.

### 2.3 Study Selection

**FIGURE 1:**
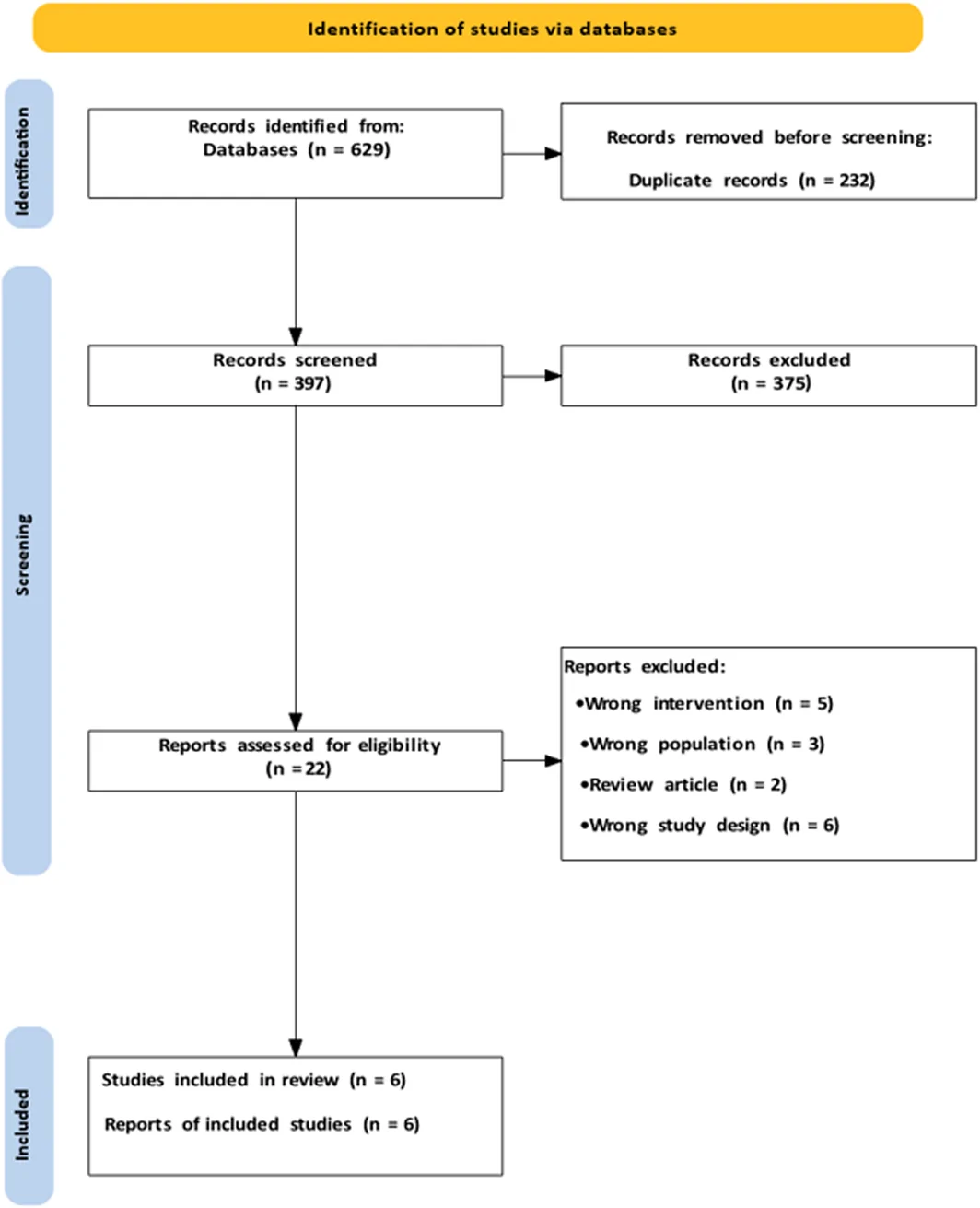
PRISMA 2020 flow diagram of study selection process.

Records from database searches were exported into the Rayyan software for management of the study selection process [22]. Once duplicates were removed, two independent reviewers (LN and RS) screened titles and abstracts against the eligibility criteria. Full‐text articles of potentially relevant records were then assessed. Any disagreements between reviewers were resolved through discussion or, if needed, by consulting a third reviewer (LH). The selection process was documented using the PRISMA flow diagram (Figure 1).

### 2.4 Data Extraction and Management

Data were extracted from included studies by two independent reviewers (LN and CG) using a piloted data extraction form. Information collected included: study characteristics (author, year, country, design, duration), participant details (sample size, age, sex), intervention specifics (components, duration, intensity) and outcome measures (hospital length of stay, postoperative complications, mortality, readmission, hospital costs). Disagreements in extraction were resolved by consensus or a third reviewer (RS) arbitration.

### 2.5 Risk of Bias and Quality Assessment

The methodological quality of included RCTs was evaluated using the Cochrane Risk of Bias tool version 2 (RoB 2) [23]. Comparative observational studies were assessed using the Newcastle‐Ottawa Scale (NOS) [24]. Two reviewers (LN and RS) conducted assessments independently. Consensus was reached through discussion.

### 2.6 Statistical Analysis

Statistical analysis was performed using Review Manager (RevMan v5.4, the Cochrane Collaboration) [25]. For dichotomous outcomes, odds ratios (ORs) with 95% confidence intervals (CIs) were calculated. For continuous outcomes, mean differences (MD) or standardized mean differences (SMD) with 95% CIs were computed. If studies reported continuous data as median and interquartile range, we converted these to mean and standard deviation using established methods [26]. Statistical heterogeneity was assessed using the *I* ^2^ statistic and the Chi^2^ test. A fixed‐effects model was used when a common effect size across studies could reasonably be assumed. Heterogeneity was interpreted using Cochrane Handbook thresholds: 0% – 40% might not be important; 30% – 60% moderate; 50% – 90% substantial; 75% – 100% considerable. A random‐effects model was applied when clinical or methodological heterogeneity suggested variation in true effects between studies. Publication bias was planned to be explored using funnel plots and Egger's test if sufficient studies (≥ 10) were included.

## 3 Results

### 3.1 Search Results

The initial search yielded 629 records. After removing duplicates and screening titles and abstracts, 22 full‐text reports were assessed for eligibility. Sixteen studies were excluded for not meeting the criteria (Supporting Information S2), and the remaining six studies were included (Figure 1).

### 3.2 Characteristics of Included Studies

Of the six included studies, four were RCTs and two were comparative cohort studies. The total sample size across all studies was 557 patients, with individual study cohorts ranging from 34 to 205 participants. Of the total sample, 299 patients were in prehabilitation groups, while 258 were in non‐prehabilitation groups. Prehabilitation interventions varied in type (unimodal, bimodal or multimodal) and duration (three studies delivered programs ≤ 4 weeks; one study delivered a 6‐week program; one study delivered a 1‐week program; one study did not specify duration). The characteristics of each included prehabilitation program are summarized in Supporting Information S3. The study by Nakajima et al. [27] included patients undergoing mixed hepato‐pancreato‐biliary (HPB) procedures and only data from the liver resection subgroup were included in the meta‐analysis. Kaibori and colleagues [15] included postoperative exercise from 1 week after surgery, continuing for 6 months; therefore, the observed effects in this study cannot be attributed to prehabilitation alone. Full details of the characteristics of the included studies are presented in Table 1. Full details of the baseline characteristics of the included studies, including BMI, nutritional status, frailty/sarcopenia, surgical approach and neoadjuvant therapy are presented in Supporting Information S4.

**TABLE 1:** Characteristics of included studies.

Author (first author's name)	Year	Country	Study design (prospective cohort study, RCT & Retrospective cohort study)	Type of surgery	Prehabilitation type (unimodal, bimodal, and multimodal)	Prehabilitation interventions (physical activity, psychological support & nutritional support)	Duration	Control group conditions	Sample size		Age (median [IQR], median [range] or mean ± SD)		Sex		NOS score (Max 9 stars)
									Prehab	Non‐prehab	Prehab	Non‐prehab	Prehab	Non‐prehab	
Berardi et al. [28]	2025	Italy	RCT	Hepatectomy	Bimodal	Physical exercise and nutritional support	6 weeks	Standard perioperative care; no prehabilitation	30	30	69 (60–75)	69 (63–74)	15:15	17:13	
Dunne et al. [14]	2016	United Kingdom	RCT	Hepatectomy	Unimodal	Physical exercise—high‐intensity cycle & interval training program	4 weeks (12 sessions) pre‐operatively	Standard care; no exercise advice	20	17	61 (56–66)	62 (53–72)	13:7	13:4	
Kaibori et al. [15]	2013	Japan	RCT	Hepatectomy	Unimodal	Physical exercise—exercise at the anaerobic threshold	1 month pre‐operatively and 6 months post‐operatively (perioperative)	Diet therapy alone; no exercise	25	26	68.0 ± 9.1	71.36 ± 8.8	17:8	19:7	
Liang et al. [29]	2025	China	RCT	Hepatectomy	Unimodal	Physical exercise—aerobic, resistance exercises & respiratory training	1 week	Routine clinical care; no exercise	104	101	57.69 ± 11.4	53.25 ± 9.7	88:16	78:23	
Nakajima et al. [27]	2019	Japan	Retrospective cohort study	Hepatectomy, pancreatoduodenectomy & hepato‐pancreatoduodenectomy	Bimodal	Physical exercise and nutritional support	Not stated	Historical controls; no prehabilitation	51	52	69 (65–76)	69 (60–75)	51:25	53:23	7
Wang et al. [30]	2020	Singapore	Prospective cohort study	Hepatectomy	Multimodal	Physical activity—breathing exercises, individualized exercise program Nutritional support—dietitian counseling & oral supplements Psychological support—counseling for anxiety & discharge planning	2–4 weeks (minimum 2 weeks) pre‐operatively	Standard care (anesthesia + financial counseling)	70	34	68 (40–80)	66 (46–87)	52:18	25:9	6

### 3.3 Risk of Bias Assessment of Included Studies

**FIGURE 2:**
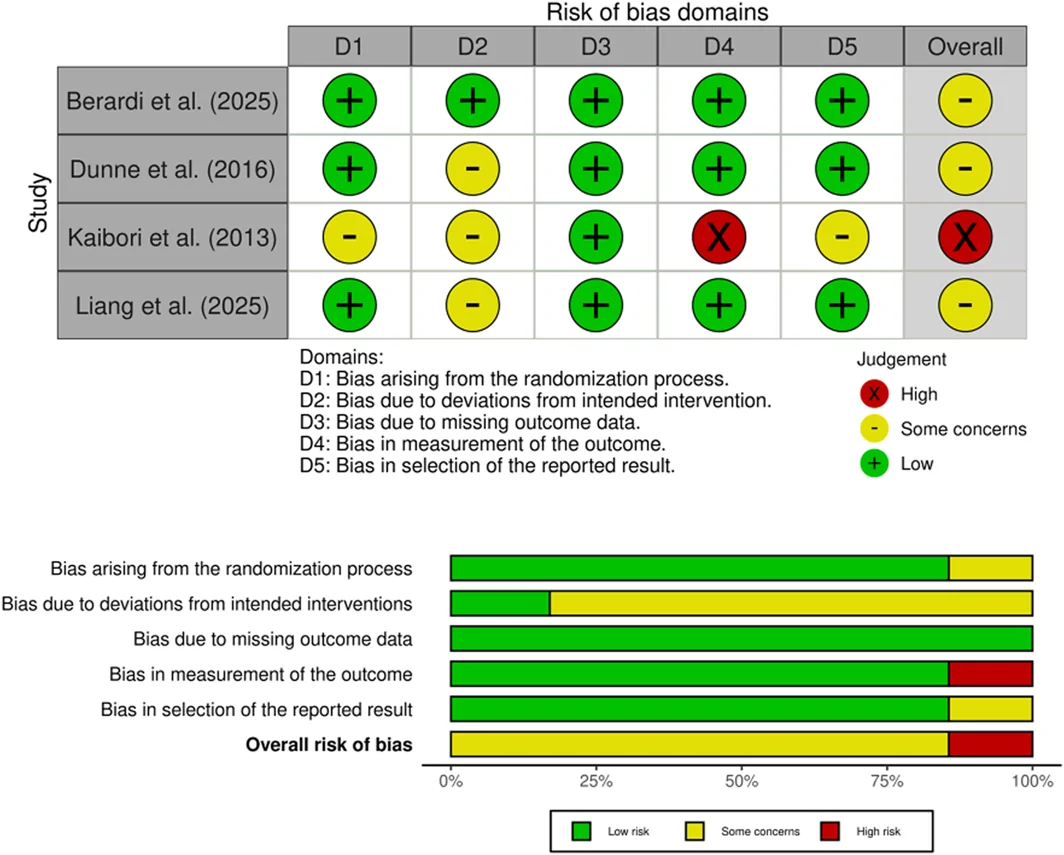
Risk of bias summary for included randomized controlled trials (RoB 2 tool).

The methodological quality of the four RCTs was assessed using the Cochrane Risk of Bias tool version 2 (RoB 2) [23]. Overall, the RCTs were assessed as having some concerns across key domains, particularly due to the lack of blinding of participants and personnel. A summary of the risk of bias assessment for the included RCTs is presented in Figure 2. The methodological quality of the two comparative observational studies was assessed using the Newcastle‐Ottawa Scale (NOS) [24]. Both studies were rated as moderate quality, with scores of 6 and seven out of a possible nine points. The detailed NOS domain‐level ratings for the observational studies are provided in Supporting Information S5.

### 3.4 Pooled Outcomes

#### 3.4.1 Hospital Length of Stay

**FIGURE 3:**
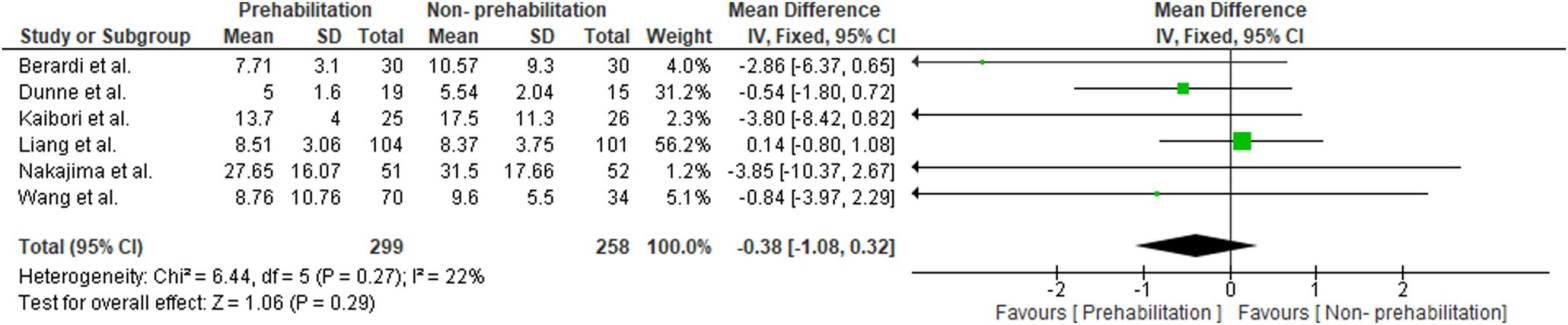
Forest plot of length of hospital stay (days).

All studies reported the hospital length of stay. The pooled analysis showed no statistically significant difference between the prehabilitation and non‐prehabilitation groups (Mean Difference [MD] = −0.38 days; 95% Confidence Interval [CI], −1.08 to 0.32; *p* = 0.29). Heterogeneity among the studies was low (*I* ^2^ = 22%; *p* = 0.27) (Figure 3). Mean hospital length of stay across studies ranged from 5.2 to 12.1 days in the prehabilitation groups and from 5.8 to 12.5 days in the control groups.

#### 3.4.2 Overall Complication Rates

**FIGURE 4:**
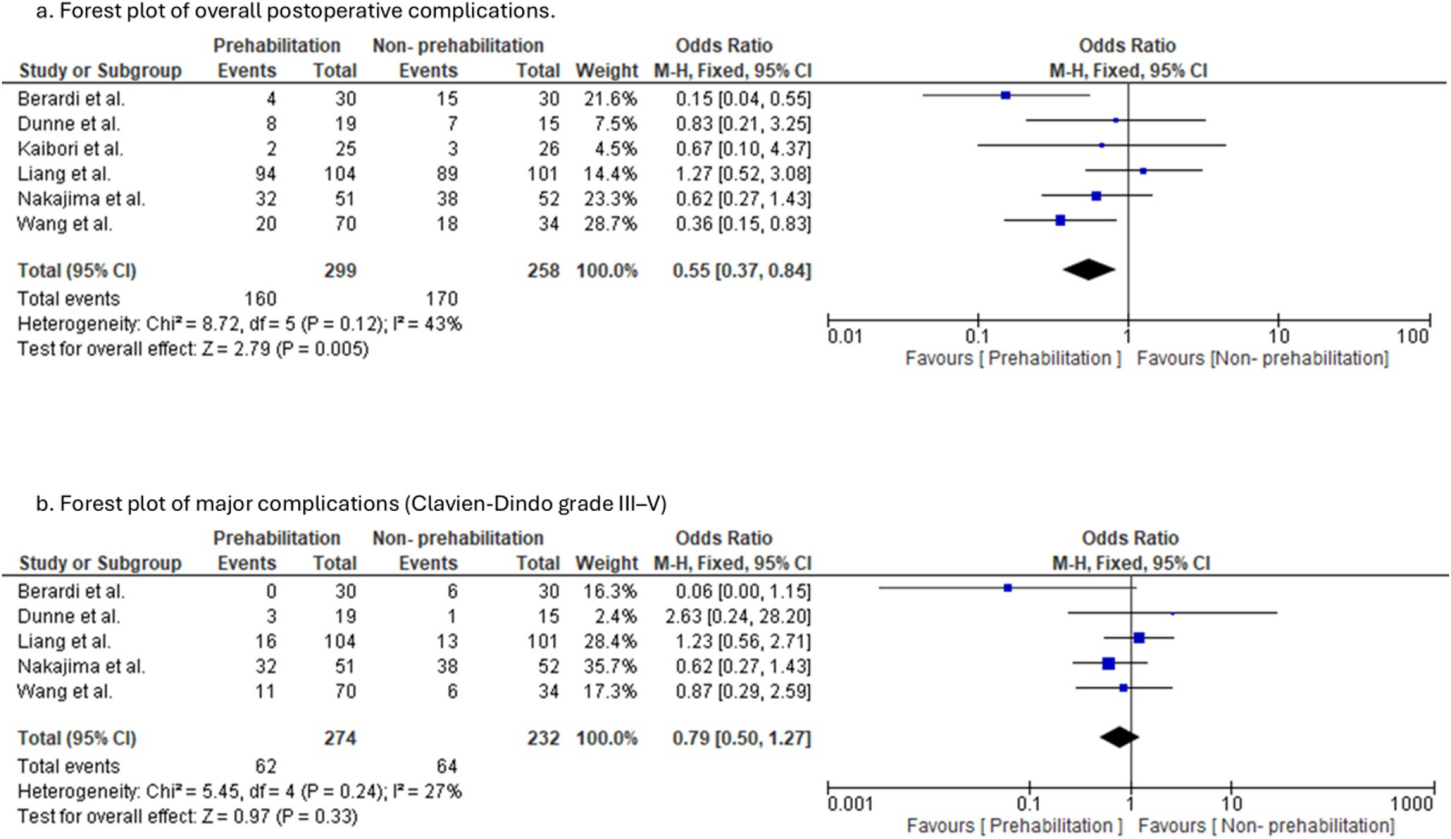
(a) Forest plot of overall postoperative complications & (b) Forest plot of major complications (Clavien‐Dindo grade III–V).

All studies evaluated overall postoperative complications. The prehabilitation group experienced fewer overall complications compared to the non‐prehabilitation group (Odds Ratio [OR] = 0.55; 95% CI, 0.37 to 0.84; *p* = 0.005). Heterogeneity was moderate (*I* ^2^ = 43%; *p* = 0.12) (Figure 4a).

#### 3.4.3 Major Complications

Data on major complications were available from five studies (506 patients). Nakajima et al. [27] recorded 32 events in the prehabilitation group (*n* = 51) and 38 events in the non‐prehabilitation group (*n* = 52), which accounted for 35.7% of the weight in the meta‐analysis. The incidence of major complications ranged from 0% to 62.7% in the prehabilitation groups and 6.7%–73.1% in the non‐prehabilitation groups. One study [28] reported no major complications in the prehabilitation group. The pooled analysis found no statistically significant difference in major complication rates between groups (OR = 0.79; 95% CI, 0.50 to 1.27; *p* = 0.33). Heterogeneity was low (*I* ^2^ = 27%; *p* = 0.24) (Figure 4b).

#### 3.4.4 Mortality

**FIGURE 5:**
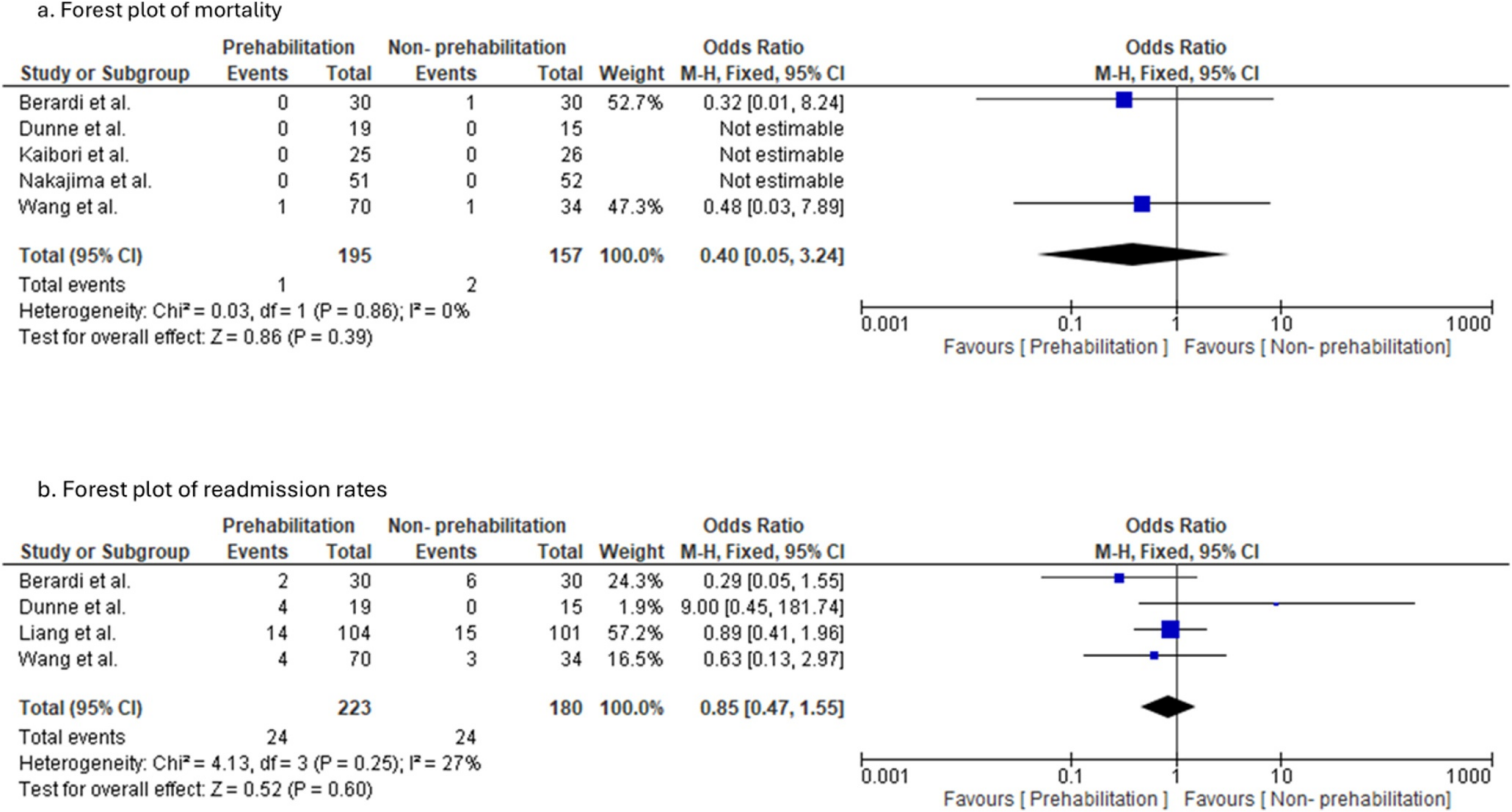
(a) Forest plot of mortality & (b) Forest plot of readmission rates.

Five studies reported mortality data (352 patients). The studies by Berardi et al. [28] and Kaibori et al. [15] reported no mortality events in either group. Only three mortality events occurred in total (one in prehabilitation vs. two in non‐prehabilitation groups). The pooled analysis showed no significant difference between groups (OR = 0.40; 95% CI, 0.05 to 3.24; *p* = 0.39), with no observed heterogeneity (*I* ^2^ = 0%; *p* = 0.86) (Figure 5a).

#### 3.4.5 Readmission Rates

Four studies provided data on hospital readmissions (403 patients). The incidence of readmissions ranged from 5.7% to 21.1% in the prehabilitation groups and from 0% to 20% in the non‐prehabilitation groups. There was no significant difference found between the prehabilitation and non‐prehabilitation groups (OR = 0.85; 95% CI, 0.47 to 1.55; *p* = 0.60). Heterogeneity was low (*I* ^2^ = 27%; *p* = 0.25) (Figure 5b).

#### 3.4.6 Hospital Costs

**FIGURE 6:**
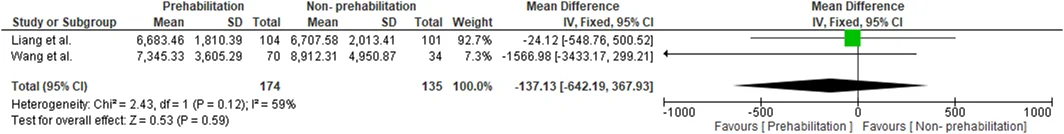
Forest plot of hospital costs (US dollars).

Only two studies reported on hospitalization costs (309 patients). The pooled mean difference in costs was not statistically significant (MD = −137.13; 95% CI, −642.19, 367.93; *p* = 0.59). Heterogeneity was substantial but not statistically significant (*I* ^2^ = 59%; *p* = 0.12) (Figure 6).

## 4 Discussion

This systematic review and meta‐analysis provides a procedure‐specific evaluation of the impact of prehabilitation on clinical and economic outcomes in patients undergoing liver resection. This review found that prehabilitation was associated with fewer overall postoperative complications following liver resection. However, the pooled analysis included both randomized and observational data, and the effect size should be interpreted with appropriate caution. There was no statistically significant effect demonstrated on length of stay, major complications, mortality and readmission, or the first pooled analysis of hospitalization costs. These findings suggest a procedure‐specific effect profile, demonstrating that prehabilitation can enhance general postoperative resilience but is less effective at influencing endpoints largely driven by intraoperative, anatomical or disease‐related factors in liver surgery.

The findings confirm that prehabilitation did not significantly reduce length of hospital stay (LOS), a result consistent with recent HPB meta‐analyses such as Lubbad et al. [19] and earlier work by Dagorno et al. [16]. This contrasts with the significant reduction reported by Deprato et al. [17], a finding that may have been swayed by the inclusion of surgical cohorts with recovery trajectories more responsive to preoperative optimization. Notably, length of stay is a complex endpoint often influenced by baseline fitness. Most patients undergoing liver resection are over 60 years old and many have poor functional reserve [31, 32]. A short prehabilitation program may not achieve meaningful fitness optimization in this group. Furthermore, length of stay may also be impacted by institutional practices, healthcare system incentives and cultural factors that vary widely across settings [33, 34]. For example, discharge practices differ considerably between healthcare systems, with strong policy incentives for early discharge in publicly funded systems such as the UK [33], whereas financial structures in some private healthcare settings may reduce incentives for shorter hospital stays [34]. These systemic influences may limit the ability of prehabilitation to demonstrate an effect on LOS, even when functional recovery is enhanced. The unique physiological and procedural demands of liver surgery, such as postoperative hepatic regeneration, biliary management and perioperative fluid and electrolyte dynamics, may also limit the effect of prehabilitation on LOS. Thus, while prehabilitation enhances perioperative resilience, its impact on length of stay in liver resection is modest, highlighting the need for procedure‐specific evaluation of HPB outcomes and greater emphasis on patient‐centered measures such as functional recovery.

The reduction in overall postoperative complications is clinically relevant and aligns with the conclusions of Lubbad et al. [19]. This finding represents meaningful progress from earlier broad reviews, such as Dewulf et al. [18], which found only a non‐significant trend toward fewer complications. The convergence of evidence from our liver‐specific synthesis and the recent HPB meta‐analysis by Lubbad et al. [19] reinforces the view that prehabilitation provides a morbidity‐reducing benefit in patients undergoing liver resection. Crucially, the consistent lack of effect on major complications across all reviews, including this present review, suggests prehabilitation may improve physiological reserve but does not prevent serious surgical complications. The benefit of prehabilitation may be limited in certain high‐risk procedures. Patients with hilar cholangiocarcinoma often require bile duct excision and resection of additional organs [35]. Patients with gallbladder cancer similarly require extensive resection. By contrast, minor non‐anatomical resections for colorectal liver metastases carry a lower risk profile. Patients requiring pre‐operative optimization, such as biliary drainage or liver augmentation procedures, represent another high‐risk group. These complex operations carry high complication rates that may not be modifiable by preoperative optimization alone. Prehabilitation may help build physiological resilience to a certain extent [36], but its effect may be limited in the context of major liver resection. This distinction between systemic and technical outcomes is a key insight afforded by a procedure‐specific analytical approach.

An additional contribution of this review is the inclusion of an economic analysis, which is lacking in previous HPB meta‐analyses. We attempted to pool cost data, but only two studies reported cost outcomes. The pooled estimate was not statistically significant and heterogeneity was substantial. These findings should be considered preliminary. Nevertheless, this represents an initial step toward evaluating the economic impact of prehabilitation in liver surgery, an increasingly measured outcome in perioperative care pathways. Future research should include a standardized cost analysis that accounts for both direct and indirect costs and incorporates a broader range of outcomes, such as antibiotic therapy for sepsis, radiological interventions, intensive care or high‐dependency unit stays, further imaging, reoperation and discharge to nursing homes or rehabilitation facilities. However, cost is generally difficult to calculate: a complexity that should be addressed in future study designs. In the absence of robust and sufficient cost data, the economic benefit of prehabilitation in liver resection remains unclear, while its clinical benefit of reducing overall complications supports the need for further investment in structured prehabilitation programs.

A notable finding from this analysis is the significant variation in prehabilitation intervention design of the included studies. The interventions implemented across the studies ranged from unimodal physical exercise to multimodal interventions that incorporated physical activity, nutritional optimization and psychological support. The program's duration also varied, ranging from 1 week to more than 4 weeks, with no clear justification provided for timing or intensity. This variation is particularly pertinent to liver surgery, where patient‐specific factors, including pre‐existing metabolic dysfunction, sarcopenia and typically restricted preoperative timelines, hinder program implementation and compliance.

Despite this heterogeneity, the finding that prehabilitation was associated with reduced complications is noteworthy. That a benefit emerged across diverse programs strengthens the case for integrating prehabilitation into standard care and supports investment by clinicians and commissioners. The lack of standardized, liver‐specific prehabilitation interventions limits the ability to determine effective program design, reinforcing the need for tailored, feasibility‐informed protocols in this cohort.

Delivering prehabilitation in routine practice presents additional challenges. Many patients are referred from other regions to specialist centers, making in‐person attendance difficult and potentially reducing engagement. While home‐based or virtual programs are offered as a practical alternative, they often encounter barriers, including travel cost, distance and time [37]. Low adherence is another challenging variable. In Liang et al.’s [29] study, only 54% of patients adhered to the prescribed program. Virtual supervision may improve adherence compared with unsupervised home‐based programs; for example, an app‐based prehabilitation program with health coach support achieved a 76.3% engagement rate [37]. However, such programs require appropriate infrastructure and patient digital literacy. These practical considerations must be addressed when designing implementation strategies for prehabilitation in liver surgery.

### 4.1 Limitations

Despite being a dedicated meta‐analysis of prehabilitation in liver resection, several limitations should be considered. The analysis is constrained by the predominance of small, single‐center studies and heterogeneous designs, which limits the generalizability of the findings. Key liver‐specific outcomes, particularly post‐hepatectomy volumetric and functional recovery, were rarely reported across the included studies, restricting evaluation of the effect of prehabilitation on liver regeneration. Hospitalization cost data were only provided in two studies, and patient‐reported recovery outcome measures were similarly scarce, limiting a comprehensive cost‐effectiveness evaluation. Finally, most included studies were conducted in high‐income settings, which may limit applicability to lower‐resource healthcare settings.

## 5 Conclusion

This systematic review and meta‐analysis provide a comprehensive evaluation of prehabilitation in patients undergoing liver resection. The key finding is that prehabilitation significantly reduces overall postoperative complications. However, no significant effects were found for length of stay, major complications, mortality, readmission or hospitalization costs. This may suggest a procedure‐specific effect profile whereby prehabilitation enhances general postoperative resilience but does not influence endpoints largely driven by intraoperative, anatomical or disease‐related factors. Significant variation in prehabilitation intervention design limits the ability to determine effective program structure, reinforcing the need for standardized, liver‐specific protocols. The economic benefit remains unclear due to insufficient cost data. Furthermore, critical gaps remain in the reporting of liver‐specific endpoints, including post‐hepatectomy functional recovery and patient‐reported outcomes, which merit further investigation.

## Author Contributions

**Lyrics M. Noba:** conceptualization, investigation, methodology, validation, writing – review and editing, formal analysis, visualization, writing – original draft, project administration. **Robyn J. Stiger:** conceptualization, writing – review and editing, investigation, methodology, validation. **Declan F. J. Dunne:** conceptualization, writing – review and editing, supervision, validation. **Lawrence D. Hayes:** conceptualization, writing – review and editing, methodology, validation. **Christopher J. Gaffney:** conceptualization, writing – review and editing, methodology, validation. **Ramani S. Moonesinghe:** conceptualization, writing – review and editing, supervision, validation. **Ewen M. Harrison:** conceptualization, writing – review and editing, supervision, validation.

## Funding

The authors have nothing to report.

## Ethics Statement

The authors have nothing to report.

## Conflicts of Interest

The authors declare no conflicts of interest.

## Supporting information


Supporting Information S1

Supporting Information S2

Supporting Information S3

Supporting Information S4

Supporting Information S5

## Data Availability

The authors have nothing to report.
