# Supplementary material for: The Impact of Prehabilitation on Clinical Outcomes in Liver Surgery: A Systematic Review and Meta‐Analysis
Source: World J Surg. 2026 Jul 20;50(9):2704–13. doi: 10.1002/wjs.70501 (PMC13576545; doi:10.1002/wjs.70501)
Supplement: Supplementary file 1 [file WJS-50-2704-s001.docx]

**Newcastle-Ottawa Scale (NOS) Assessment for Risk of Bias in Included Studies**

| Study | NOS Domain: Selection (max 4★) | NOS Domain: Comparability (max 2★) | NOS Domain: Outcome (max 3★) | Total NOS Score (max 9★) | Summary of Risk of Bias |
| --- | --- | --- | --- | --- | --- |
| **Wang et al. (2020)** | ★★★ (Somewhat representative cohorts; non-exposed from the same source; secure exposure record; outcome not present at start) | ★ (Controls for age, gender, ASA, surgery type) | ★★ (Record linkage; adequate follow-up; incomplete survey data described) | 6/9 | Moderate risk. Non-randomised design with basic confounder control; baseline comorbidity imbalance (higher Charlson score in prehab group) may introduce residual bias. |
| **Nakajima et al. (2019)** | ★★★ (Somewhat representative cohorts; non-exposed from the same source; secure exposure record; outcome not present at start) | ★★ (Propensity score matching for gender, age, BMI, wait time, surgery type, albumin, lymphocyte count) | ★★ (Record linkage; adequate follow-up; complete follow-up for matched cohort) | 7/9 | Low risk. Well-matched historical control study using propensity scoring for key confounders; robust methodological approach. |
