# Supplementary material for: The Impact of Prehabilitation on Clinical Outcomes in Liver Surgery: A Systematic Review and Meta‐Analysis
Source: World J Surg. 2026 Jul 20;50(9):2704–13. doi: 10.1002/wjs.70501 (PMC13576545; doi:10.1002/wjs.70501)
Supplement: Supplementary file 2 [file WJS-50-2704-s002.docx]

| **Study (first author, year)** | **Title** | **Reason for exclusion** |
| --- | --- | --- |
| Zacharias T, et al. (2021) | Preoperative immunonutrition in liver resection – a propensity score matched case‑control analysis | Wrong intervention (nutrition only) |
| Bothe A Jr, et al. (1986) | Is there a role for perioperative nutritional support in liver resection? | Wrong intervention (nutrition only) |
| Brody F, et al. (2015) | Preoperative liver reduction utilizing a novel nutritional supplement | Wrong intervention (nutritional supplement only) |
| Reeves JG, et al. (2015) | Short‑term preoperative diet modification reduces steatosis and blood loss in patients undergoing liver resection | Wrong intervention (diet modification only) |
| Abbott S, et al. (2023) | Variation and outcomes of liver‑reducing dietary regimens before bariatric surgery | Wrong intervention (diet modification only) |
| Benmassaoud A, et al. (2022) | Prehabilitation in patients awaiting liver transplantation | Wrong population (liver transplant) |
| Magalhães CBA, et al. (2021) | Exercise Capacity and Respiratory Profile in Patients after Orthotopic Liver Transplantation | Wrong population (liver transplant) |
| Plank LD, et al. (2005) | Pre‑ and postoperative immunonutrition in patients undergoing liver transplantation: a pilot study of safety and efficacy | Wrong population (liver transplant) |
| Heger P, et al. (2017) | A Systematic Review and Meta‑analysis of Physical Exercise Prehabilitation in Major Abdominal Surgery (PROSPERO 2017 CRD42017080366) | Review article |
| van Wissen J, et al. (2021) | Preoperative Methods to Reduce Liver Volume in Bariatric Surgery: a Systematic Review | Review article |
| Duarte‑Rojo A, et al. (2024) | Use of a Mobile‑Assisted Telehealth Regimen to Increase Exercise in Transplant Candidates: A Home‑Based Prehabilitation Pilot and Feasibility Trial | Wrong study design (no comparator) |
| Wijma AG, et al. (2023) | Cardiac and intramuscular adaptations following short‑term exercise prehabilitation in unfit patients scheduled to undergo hepatic or pancreatic surgery: study protocol of a multinuclear MRI study | Wrong study design (study protocol) |
| Benmassaoud A, et al. (2023) | Prehabilitation in patients with cirrhosis awaiting liver transplantation: protocol of a feasibility study | Wrong study design (study protocol) |
| AlShahrani AN, et al. (2021) | Impact of Pre- and Post-therapeutic Exercises in Sarcopenia and Pain in Liver Transplant Patients | Wrong study design (no comparator, single‑arm) |
| Case BC, et al. (2024) | Pre‑Operative Cardiovascular Testing before Liver Transplantation | Wrong study design (not a prehabilitation intervention) |
| Tanaka S, et al. (2021) | Prospective study of objective physical activity and quality of life in living donor liver transplant recipients | Wrong study design (observational, no intervention comparator) |
