# Supplementary material for: The Impact of Prehabilitation on Clinical Outcomes in Liver Surgery: A Systematic Review and Meta‐Analysis
Source: World J Surg. 2026 Jul 20;50(9):2704–13. doi: 10.1002/wjs.70501 (PMC13576545; doi:10.1002/wjs.70501)
Supplement: Supplementary file 3 [file WJS-50-2704-s003.docx]

Supplementary Table 2: Summary of Prehabilitation Programme Components and Adherence

| Study | Type | Duration | Frequency | Intensity | Supervision | Adherence | Completion Rate | Nutritional Component | Psychological Component |
| --- | --- | --- | --- | --- | --- | --- | --- | --- | --- |
| Kaibori et al. (2013) | Unimodal | 1 month pre + 6 months post | 3/week (60 min) | AT-based; CPET-determined | Unsupervised (home-based); monthly doctor/trainer confirmation | Not reported; some discontinued | Not reported | Dietary guidance (25–30 kcal/kg; protein 1.0–1.2 g/kg); dietitian monitoring | Not specified |
| Dunne et al. (2016) | Unimodal | 4 weeks | 3/week | HIIT: moderate (<60% VO₂ peak) + vigorous (>90% VO₂ peak) | Supervised (hospital exercise lab) | 95% | 95% | None | Not specified |
| Nakajima et al. (2019) | Bimodal | ~4 weeks (median 32 days) | ≥3/week (once daily) | Moderate (Borg 3–4) aerobic + resistance (2 sets of 10 reps) | Unsupervised (home-based) | Not reported | 70% | Leucine-rich essential amino acid supplement (Amino L40) | Not specified |
| Wang et al. (2020) | Multimodal | 2–4 weeks | Walking: 5×/week; Breathing: ≥4×/day | Not specified | Unsupervised (home-based) | Not formally monitored | Not reported | Individualised nutrition intervention; dietitian counselling; nutritional supplements | Case manager counselling; anxiety reduction; discharge planning; social support |
| Berardi et al. (2025) | Bimodal | 6 weeks | Daily walking + 2×/week supervised sessions | Brisk walking; supervised strength/aerobic | Mixed: unsupervised home-based + supervised in-hospital | 100% | 100% | BCAA + immune nutritional supplementation (Nitronat Plus) | Not specified |
| Liang et al. (2025) | Unimodal | 1 week (median 8 days) | Daily (≤1 hour/day) | Moderate (50–75% HR max; Borg 13–16) | Unsupervised (home-based); WeChat reminders | 54% | 54% | None | Routine guidance only |
