# Supplementary material for: The Impact of Prehabilitation on Clinical Outcomes in Liver Surgery: A Systematic Review and Meta‐Analysis
Source: World J Surg. 2026 Jul 20;50(9):2704–13. doi: 10.1002/wjs.70501 (PMC13576545; doi:10.1002/wjs.70501)
Supplement: Supplementary file 4 [file WJS-50-2704-s004.docx]

**Baseline Characteristics of Included Studies**

| **Author** | **BMI** | | **Nutritional Status** | | **Frailty/Sarcopenia** | | **Surgical Approach** | | **Neoadjuvant Therapy** | |
| --- | --- | --- | --- | --- | --- | --- | --- | --- | --- | --- |
|  | **Prehab** | **Non-Prehab** | **Prehab** | **Non-Prehab** | **Prehab** | **Non-Prehab** | **Prehab** | **Non-Prehab** | **Prehab** | **Non-Prehab** |
| **Berardi et al. [27]** | 24.6 (23.0–26.5) | 24.0 (23.0–26.0) | Not reported | Not reported | Sarcopenia (SMI + handgrip) – inclusion criterion | Sarcopenia (SMI + handgrip) – inclusion criterion | 33.3% laparoscopic | 33.3% laparoscopic | Not reported | Not reported |
| **Dunne et al. [14]** | 29.7 (4.2) | 29.3 (4.2) | Not reported | Not reported | High-risk (VO₂ AT <11): 9/20 | High-risk (VO₂ AT <11): 8/17 | Not specified | Not specified | 55% (11/20) | 41.2% (7/17) |
| **Kaibori et al. [15]** | Not reported | Not reported | Body mass: 97 ± 4% baseline; Fat mass: 98 ± 16% baseline | Body mass: 93 ± 6% baseline; Fat mass: 80 ± 15% baseline | Child-Pugh A/B (24/1) | Child-Pugh A/B (25/1) | Not specified | Not specified | Not reported | Not reported |
| **Liang et al. [28]** | 23.51 (3.43) | 23.42 (3.54) | Normal | Normal | Not reported | Not reported | 60.6% laparoscopic; 39.4% open | 60.4% laparoscopic; 39.6% open | Not reported | Not reported |
| **Nakajima et al. [26]** | 22.2 (20.4–23.7) | 21.4 (19.6–23.7) | PNI 43.8 (40.1–48.5); albumin 3.7 (3.4–4.1) | PNI 45.5 (42.1–50.4); albumin 4.0 (3.6–4.3) | Not reported | Not reported | Open only | Open only | None (excluded) | None (excluded) |
| **Wang et al. [29]** | 23.66 (15.67–40.49) | 23.13 (16.8–33.71) | Serum albumin 39 (21–46) g/dL; SGA | Serum albumin 38 (16–48) g/dL; SGA | Not reported | Not reported | 35.7% laparoscopic; 64.3% open | 29.4% laparoscopic; 70.6% open | 30.0% (21/70) | 26.5% (9/34) |
