# Supplementary material for: The Impact of Prehabilitation on Clinical Outcomes in Liver Surgery: A Systematic Review and Meta‐Analysis
Source: World J Surg. 2026 Jul 20;50(9):2704–13. doi: 10.1002/wjs.70501 (PMC13576545; doi:10.1002/wjs.70501)
Supplement: Supplementary file 5 [file WJS-50-2704-s005.docx]

UPDATED SEARCH STRATEGIES — LIVER SURGERY

**MEDLINE / EMBASE (Ovid)**

1 — Population (Liver Surgery)

(exp Liver/su or exp Hepatectomy/ or (liver* adj3 (resect* or surg* or operat* or lobectom* or hepatectom*)).ti,ab,kf. or hepatectom*.ti,ab,kf. or "hepatic surgery".ti,ab,kf. or "liver surgery".ti,ab,kf.)

2 — Core Prehab Terms

(prehab* or pre-hab* or pre-rehab* or "pre rehab*" or "preoperative exercise therapy".ti,ab,kf. or "preoperative programme".ti,ab,kf. or "preoperative program".ti,ab,kf. or "preoperative exercise".ti,ab,kf.)

3 — Preoperative Care Context

(exp Preoperative Care/ or (preoperative or pre-operative or preop).ti,ab,kf.)

4 — Physical Component

(exp Exercise/ or (exercise* or physical activ* or resistance train* or physiotherapy or "respiratory training" or "inspiratory muscle training" or "breathing exercise*").ti,ab,kf.)

5 — Nutrition Component

exp Nutritional Support/ or (nutrition* adj3 (support* or optimi* or therap* or interven*)).ti,ab,kf. or immunonutrition.ti,ab,kf. or ONS.ti,ab,kf. or "oral nutritional supplement*".ti,ab,kf. or "preoperative nutrition".ti,ab,kf. or "preoperative feeding".ti,ab,kf. or "enteral nutrition".ti,ab,kf. or "preoperative carbohydrate loading".ti,ab,kf.

6 — Psychology Component

exp Psychotherapy/ or ((psycholog* adj3 (support* or therap*)) or CBT or mindfulness or "anxiety reduction" or "stress management" or "behaviour change" or counselling or "motivational interviewing" or psychoeducation).ti,ab,kf.

7 — Combine Intervention

(2 or (3 and (4 or 5 or 6)))

8 — Study Designs

(exp Randomized Controlled Trial/ or exp Cohort Studies/ or (randomized or randomised or cohort or prospective or comparative or retrospective).ti,ab,kf.)

9 — Final Combination

(1 and 7 and 8)

**WEB OF SCIENCE**

(liver* NEAR/3 (resect* OR surg* OR operat* OR lobectom* OR hepatectom*) OR hepatectom* OR "hepatic surgery" OR "liver surgery")

(prehab* OR "pre-hab*" OR "pre-rehab*" OR "pre rehab*" OR "preoperative exercise therapy" OR "preoperative programme" OR "preoperative program" OR "preoperative exercise")

("Preoperative Care" OR preoperative OR "pre-operative" OR preop)

(exercise* OR "physical activ*" OR "resistance train*" OR physiotherapy OR "respiratory training" OR "inspiratory muscle training" OR "breathing exercise*")

("nutrition* support" OR "nutrition* therap*" OR "nutrition* interven*" OR immunonutrition OR "oral nutritional supplement*" OR ONS OR "preoperative nutrition" OR "preoperative feeding" OR "enteral nutrition" OR "preoperative carbohydrate loading")

("psycholog* support" OR "psycholog* therap*" OR CBT OR mindfulness OR "anxiety reduction" OR "stress management" OR "behaviour change" OR counselling OR "motivational interviewing" OR psychoeducation)

#2 OR (#3 AND (#4 OR #5 OR #6))

(randomized OR randomised OR "randomly assigned" OR "clinical trial" OR RCT)

(cohort OR prospective OR retrospective OR comparative OR "case control" OR observational)

#1 AND #7 AND (#8 OR #9)

**PUBMED**

("Liver/surgery"[Mesh] OR "Hepatectomy"[Mesh] OR (liver*[tiab] AND (resect*[tiab] OR surg*[tiab] OR operat*[tiab] OR lobectom*[tiab] OR hepatectom*[tiab])) OR hepatectom*[tiab] OR "hepatic surgery"[tiab] OR "liver surgery"[tiab])

AND

("Preoperative Care"[Mesh] OR "Preoperative Period"[Mesh] OR prehab*[tiab] OR "pre-hab*"[tiab] OR "pre-rehab*"[tiab] OR "pre rehab*"[tiab] OR preoperative[tiab] OR pre-operative[tiab] OR preop[tiab] OR "preoperative exercise therapy"[tiab] OR "preoperative programme"[tiab] OR "preoperative program"[tiab] OR "preoperative exercise"[tiab])

AND

("Exercise"[Mesh] OR "Exercise Therapy"[Mesh] OR "Nutritional Support"[Mesh] OR "Diet Therapy"[Mesh] OR "Psychotherapy"[Mesh] OR exercise*[tiab] OR "physical activ*"[tiab] OR "resistance train*"[tiab] OR physiotherapy[tiab] OR "respiratory training"[tiab] OR "inspiratory muscle training"[tiab] OR "breathing exercise*"[tiab] OR "nutrition* support"[tiab] OR immunonutrition[tiab] OR ONS[tiab] OR "oral nutritional supplement*"[tiab] OR "preoperative nutrition"[tiab] OR "preoperative feeding"[tiab] OR "enteral nutrition"[tiab] OR "preoperative carbohydrate loading"[tiab] OR "psycholog* support"[tiab] OR CBT[tiab] OR mindfulness[tiab] OR "anxiety reduction"[tiab] OR "stress management"[tiab] OR "behaviour change"[tiab] OR counselling[tiab] OR "motivational interviewing"[tiab] OR psychoeducation[tiab])

AND

("Randomized Controlled Trial"[pt] OR "Controlled Clinical Trial"[pt] OR randomized[tiab] OR randomised[tiab] OR randomly[tiab] OR trial[tiab] OR "Clinical Trial"[pt] OR "Cohort Studies"[Mesh] OR cohort[tiab] OR prospective[tiab] OR retrospective[tiab] OR comparative[tiab] OR "case control"[tiab] OR observational[tiab])

**COCHRANE LIBRARY**

1. hepatectomy OR liver near/3 (resect* OR surg* OR operat* OR lobectomy) OR "hepatic surgery" OR "liver surgery"
2. prehab* OR pre-hab OR pre-rehab OR "preoperative exercise therapy" OR "preoperative programme" OR "preoperative program" OR "preoperative exercise"
3. preoperative OR pre-operative OR preop
4. exercise* OR physical activ* OR resistance train* OR physiotherapy OR "respiratory training" OR "inspiratory muscle training" OR "breathing exercise"
5. nutrition* support OR immunonutrition OR ONS OR "oral nutritional supplement" OR "preoperative nutrition" OR "preoperative feeding" OR "enteral nutrition" OR "preoperative carbohydrate loading"
6. psychology* support OR CBT OR mindfulness OR "anxiety reduction" OR "stress management" OR "behaviour change" OR counselling OR "motivational interviewing" OR psychoeducation
7. #3 AND (#4 OR #5 OR #6)
8. #2 OR #7
9. #1 AND #8
